# Imaging in anatomy: a comparison of imaging techniques in embalmed human cadavers

**DOI:** 10.1186/1472-6920-13-143

**Published:** 2013-10-25

**Authors:** Grit Gesine Ruth Schramek, Dietrich Stoevesandt, Ansgar Reising, Jan Thomas Kielstein, Marcus Hiss, Heike Kielstein

**Affiliations:** 1 Department of Anatomy and Cell Biology, Martin Luther University of Halle-Wittenberg, Magdeburger Str. 8, 06108 Halle (Saale), Germany; 2 Diagnostic Radiology, Faculty of Medicine, Martin Luther University of Halle-Wittenberg, Magdeburger Str. 8, 06108 Halle (Saale), Germany; 3 Department of Internal Medicine, Division of Nephrology and Hypertension, Medical School Hannover, Carl-Neuberg-Str. 1, 30625 Hannover, Germany

**Keywords:** Medical education, Teaching, Ultrasound, Magnetic resonance imaging, Computed tomography, X-ray, Imaging

## Abstract

**Background:**

A large variety of imaging techniques is an integral part of modern medicine. Introducing radiological imaging techniques into the dissection course serves as a basis for improved learning of anatomy and multidisciplinary learning in pre-clinical medical education.

**Methods:**

Four different imaging techniques (ultrasound, radiography, computed tomography, and magnetic resonance imaging) were performed in embalmed human body donors to analyse possibilities and limitations of the respective techniques in this peculiar setting.

**Results:**

The quality of ultrasound and radiography images was poor, images of computed tomography and magnetic resonance imaging were of good quality.

**Conclusion:**

Computed tomography and magnetic resonance imaging have a superior image quality in comparison to ultrasound and radiography and offer suitable methods for imaging embalmed human cadavers as a valuable addition to the dissection course.

## Background

A wide variety of imaging techniques is an integral part of modern medicine [1], as is the knowledge about indications, side effects and limitations of techniques spanning from bed-side ultrasound over high resolution computed tomography to functional magnetic resonance imaging. Over the last decades medical students increasingly learn to interpret different imaging techniques in the first years of medical school as part of the anatomical curriculum. Cornerstones of this curriculum are the 'living anatomy’ course on the one hand, and medical images and PBL (Problem-Based-Learning) on the other hand [2-4]. Introducing radiological imaging techniques into the dissection course and macroscopic anatomy classes enhances students’ long-term ability to identify anatomical structures in medical images [5-7]. Realizing this, we introduced medical images to our education in gross anatomy several years ago. So far, we are able to provide head and hip, knee and shoulder joint CT and MRI scans of our body donors, and use archived images for x-ray based techniques as well as sonography in volunteers (fellow students) in line with the 'living anatomy’ courses. While the later one is not associated with safety hazards of the scanning itself, the potential discovery of pathological conditions needing further investigation or the need to obtain an informed consent, have to be considered as critical limitations [1]. Another fact is that images of young, healthy students do not have a lot in common with the bodies of the usually elderly body donors or future patients. There are age-related changes, physiological varieties and pathologies, and we have already been able to witness that students’ fascination and motivation is increased by viewing changes of 'their’ body donor on medical images and exploring them by dissection afterwards. Therefore, we are convinced that the preparation of structures that underwent imaging just prior to anatomical dissection causes an even greater gain in knowledge than the approach mentioned above.

However, commonly used embalming fluid, containing small amounts of formalin, not only causes a significant swelling of soft tissues [4,8], but also produces artifacts, that severely deteriorate image quality [9]. Due to the controversial discussion on imaging techniques, the aim of the present study was to compare four imaging techniques in a structured approach to decide which would be suitable for the different visualization needs of embalmed human donors and could thereby offer a valuable addition to the dissection course.

## Methods

The study was approved by the ethics commission of the medical faculty of the Martin Luther University Halle-Wittenberg. Written informed consent for scientific investigations in general is given by all body donors prior to death at the Department of Anatomy and Cell Biology.

### Subjects

For the study, we examined 23 human donors (11 male, 12 female; median age 81.1 years, SD 11.7 years, range 45 years). The cadavers investigated were preserved after standard embalming techniques, using a solution containing ethanol (77%), unbuffered formalin, glycerine, and distilled water (~7%, respectively). The procedure includes intravascular embalming for 8 hours, embalming in a solution bath for six to eight weeks, and storage at 2–4°C for up to two years. The following imaging techniques have been conducted:

### Ultrasound

Renal size parameters, including renal length, width, and cortical thickness, of both kidneys of six body donors (3 male, 3 female) were evaluated by two independent and experienced sonographers. Cadavers were investigated lying in prone position with a CX 50 Ultrasound System, C5-1 Broadband Curved Array Transducer with PureWave technology, Philips, Hamburg, Germany.

### Radiography

Both an anteroposterior (AP) and lateral view of the lumbar spine and pelvis have been performed with six body donors (3 male, 3 female) to assess the lumbar vertebral bodies and the infrarenal part of the aorta. Cadavers were investigated in supine position with the Mobilett+, Siemens, Erlangen, Germany. Evaluation has been carried out by an experienced radiologist.

### Computed tomography

A whole-body scan, including head, thorax, abdomen, pelvis, shoulder and knee joints, has been performed with 12 body donors (5 male, 7 female). The system used was the Sensation64, Siemens, Erlangen, Germany. CT-images were acquired with 120 kV and 500 effmAs and primarily reconstructed in 0.6 mm slices (0.5 mm increment). Afterwards multi planar reconstructions were made in axial, sagittal and coronal orientation (5 mm slice thickness). Evaluation has been carried out by an experienced radiologist.

### Magnetic resonance imaging

Comparable to the CT-scan a whole-body scan has been taken from 4 human donors (1 male, 3 female) with the Skyra 3 T, Siemens, Erlangen, Germany. MRI images for whole body imaging were acquired as axial T1-TSE (slice thickness 6 mm, TR 680 ms, TE 12 ms), axial T2-TSE (slice thickness 4 mm, TR 7870 ms, TE 81 ms), coronal T1-TSE (slice thickness 3 mm, TR 903 ms, TE 21 ms) and sagittal T2-TSE images (slice thickness 4 mm, TR 8520 ms, TE 100 ms). MRI images for knee joint were acquired as PD-weighted TSE sequences in axial, sagittal and coronal orientation (slice thickness 2.5 mm, TR 2800 ms, TE 19 ms). Evaluation has been carried out by an experienced radiologist.

### Evaluation criteria

The four clinical authors established imaging criteria for the explored imaging techniques according to the AIUM Practice Guideline for the Performance of an Ultrasound Examination [10], the European Guidelines on Quality Criteria for Diagnostic Radiographic Images [11], and the European Guidelines on Quality Criteria for Computed Tomography [12] (Tables 1, 2, 3 and 4). Five experienced sonographers and five experienced radiologists rated the taken images. The sonographers evaluated ultrasound images, the radiologist rated x-ray, CT, and MRI images. Evaluation of the images has been carried out individually by each expert without knowledge of the other raters’ evaluation. Following De Crop et al. [13] and Benkhadra et al. [14], who have evaluated clinical images in Thiel’s embalmed cadavers before, the criteria were rated with “0” if the anatomical structure was not visible, with “1” if the structure was poorly visible, and with “2” if the anatomical structure was easily visible. The sum of scores (total raw score) has then been divided by the number of criteria used for each imaging technique to get comparable values (calculated score).

**Table 1:** Evaluation of ultrasound images

**Image quality criteria for ultrasound – kidneys**	**Rating**					**Mean**	**Range**
	**Rater 1**	**Rater 2**	**Rater 3**	**Rater 4**	**Rater 5**		
1. Visualization of long-axis length (both poles visible)	1	1	0	0	1	0.6	1
2. Visualization of transverse length	1	0	0	1	0	0.4	1
3. Visualization of renal cortex	1	1	1	1	1	1	0
4. Visualization of renal pelvis	1	1	1	1	1	1	0
Total raw score	4	3	2	3	3	3	2
Calculated score	1	0.75	0.5	0.75	0.75	*0.75*	0.5

Rating: 0 = not visible, 1 = poorly visible, 2 = easily visible.

**Table 2:** Evaluation of x-ray images

**Image quality criteria for x-ray – lumbar spine and pelvis**	**Rating**					**Mean**	**Range**
	**Rater 1**	**Rater 2**	**Rater 3**	**Rater 4**	**Rater 5**		
1. Visualization of the upper and lower plate surfaces	1	1	1	1	1	1	0
2. Visualization of the cortex and trabecular structures	1	0	1	1	1	0.8	1
3. Visualization of the aortic shape/course	0	0	1	0	1	0.4	1
4. Visualization of aortic calcifications	2	1	1	1	1	1.2	1
Total raw score	4	2	4	3	4	3.4	2
Calculated score	1	0.5	1	0.75	1	*0.85*	0.5

Rating: 0 = not visible, 1 = poorly visible, 2 = easily visible.

**Table 3:** Evaluation of CT images

**Image quality criteria for CT – whole body**	**Rating**					**Mean**	**Range**
	**Rater 1**	**Rater 2**	**Rater 3**	**Rater 4**	**Rater 5**		
1. Visualization of thoracic wall	2	2	2	2	2	2	0
2. Visualization of entire lung parenchyma	1	2	2	2	2	1.8	1
3. Visualization of heart, aorta, and vena cava	2	1	2	1	2	1.6	1
4. Visualization of parenchymal organs	1	2	2	2	2	1.8	1
5. Visualization of vertebral structures	2	2	2	2	2	2	0
6. Visualization of shoulder, hip, and knee joint	2	1	1	1	1	1.2	1
Total raw score	10	10	11	10	11	10.4	1
Calculated score	1.7	1.7	1.8	1.7	1.8	*1.74*	0.1

Rating: 0 = not visible, 1 = poorly visible, 2 = easily visible.

**Table 4:** Evaluation of MRI images

**Image quality criteria for MRI – whole body**	**Rating**					**Mean**	**Range**
	**Rater 1**	**Rater 2**	**Rater 3**	**Rater 4**	**Rater 5**		
1. Visualization of thoracic wall	2	2	2	2	2	2	0
2. Visualization of entire lung parenchyma	1	1	1	1	1	1	0
3. Visualization of heart, aorta, and vena cava	2	1	2	2	2	1.8	1
4. Visualization of parenchymal organs	2	2	2	2	2	2	0
5. Visualization of vertebral structures	1	2	2	2	2	1.8	1
6. Visualization of shoulder, hip, and knee joint	2	2	2	2	2	2	0
Total raw score	10	10	11	11	11	10.6	1
Calculated score	1.7	1.7	1.8	1.8	1.8	*1.76*	0.1

Rating: 0 = not visible, 1 = poorly visible, 2 = easily visible.

In addition to the image quality criteria we evaluated the possibility of 3D reconstruction as well as the aspect of overall feasibility, including costs and logistics, for the four conducted imaging techniques.

## Results

### Ultrasound

**Figure 1:**
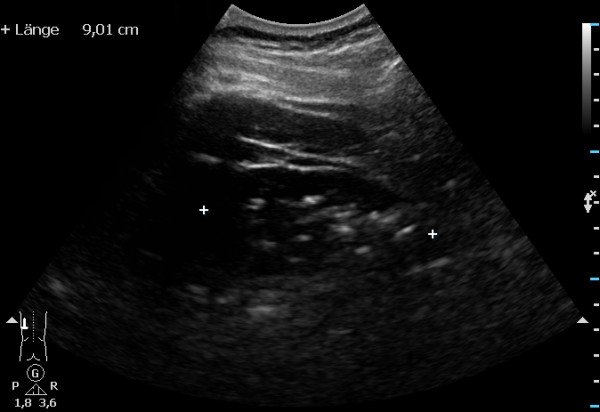
**Very poor quality of ultrasound image of the kidney.** The evaluation of reliable measurements of the kidney was unsuccessful due to severe gas artifacts.

The imaging quality of the examined kidneys was very poor (Figure 1). Although offering a non-invasive technique, severe intravascular gas artifacts, up to the small renal blood vessels (arcuate arteries) and in the surrounding tissue, were found (Additional file 1). Furthermore, in supine position some cadavers showed laminar subepidermal accumulations of gas. Tissue compression with the ultrasound transducer did not improve the imaging. The evaluation of reliable measurements of the kidneys was predominantly unsuccessful (Table 1).

### Radiography

**Figure 2:**
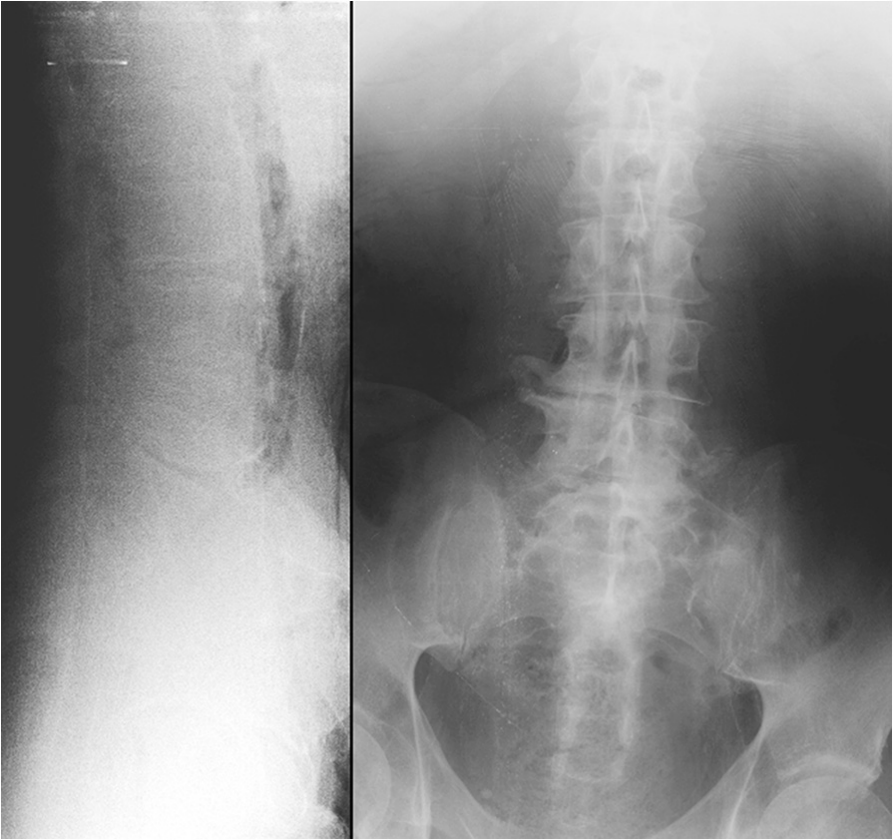
**Poor quality of lateral and AP x-ray of the lumbar spine and pelvis.** Due to the sagging of abdominal organs and additional gas artefacts in the abdominal aorta in lateral views scarcely any structures were identifiable, in AP views the lumbar spine and pelvic bones were poorly detectable.

The quality of the obtained images was poor (Table 2). The sagging of abdominal organs and additional gas artefacts in the abdominal aorta worsened the imaging quality. In AP views the lumbar spine and pelvic bones were poorly detectable, in lateral views scarcely any structures were identifiable (Figure 2).

### Computed tomography

**Figure 3:**
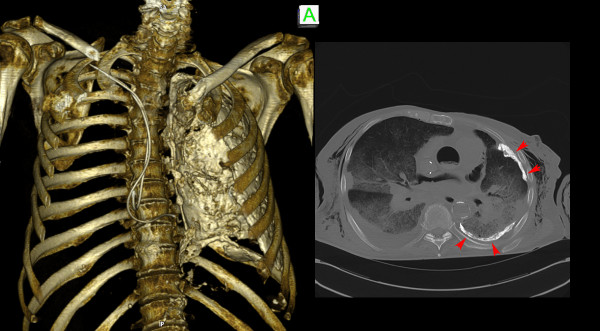
**The CT imaging quality of the skeletal system was good.** With the help of 3-D reconstruction a Pleuritis calcarea found in CT-thorax as well as the positions of pacemaker leads can be illustrated easily for teaching purposes.

The imaging quality of the skeletal system was very good (Figure 3). The scanning of other tissues was limited by subcutaneous, intravascular and intramedullary gas artefacts as well as the lack of contrast. Therefore the lungs and other parenchymal organs were scarcely assessable. The rating of the image quality criteria for CT is presented in Table 3.

### Magnetic resonance imaging

**Figure 4:**
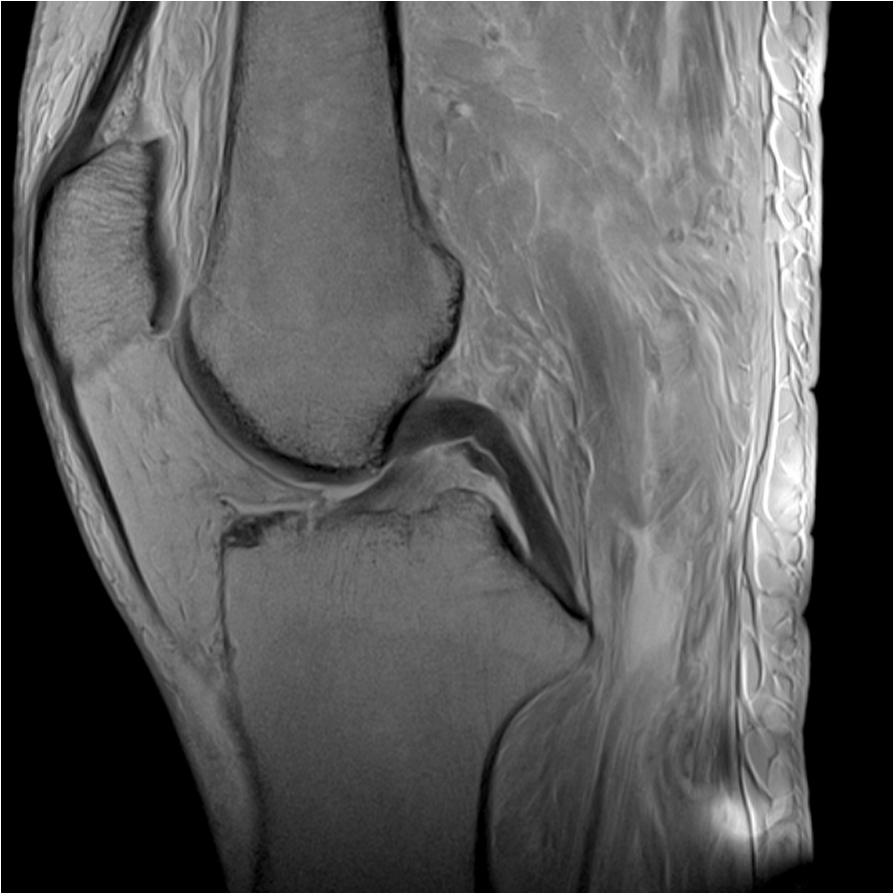
Very good quality of sagittal MRI image of the knee joint, skeletal and soft tissue structures are easily identifiable.

The quality of joint images was very good even though this imaging technique was limited by an altered signal behaviour, especially of the subcutaneous tissue (Figure 4). Table 4 shows the evaluation results for the MRI images.

In addition to the image quality criteria we evaluated the possibility of 3D reconstruction as well as the aspect of overall feasibility, including costs and logistics, for the four conducted imaging techniques. Results are shown in Table 5.

**Table 5:** Evaluation of 3D reconstruction and feasibility of imaging techniques

	**US**	**X-ray**	**CT**	**MRI**
3D reconstruction possible	0	0	2	1
Price	2	2	0	0
Logistics	2	1	0	0
Overall feasibility	2	1	0	0
Total raw score	6	4	2	1
Calculated score	1.5	1	0.5	0.25

Rating: 2 = excellent, 1 = good, 0 = poorly; for price: 2 = low expense, 0 = high expense.

## Discussion

The positive effects of introducing radiological images into the dissection course have already been described by several authors. Not only it enhances the students’ learning effects in anatomy but it also increases the overall understanding of medical imaging, thus improving the students’ long-term ability to identify anatomical structures both in vivo and in medical images [5-7,9,15]. Based on these encouraging reports, we have introduced medical images to our education in gross anatomy several years ago. So far, we are using ultrasound examinations in line with the 'living anatomy’ seminars and provide head and hip, knee and shoulder joint CT and MRI scans of our body donors. Based on our own experiences, we agree with the findings of Chew et al. [9] and Bohl et al. [15] that combining post-mortem radiological images and anatomical dissection of the same body donor leads to a greater gain in knowledge than using real patient images that are not comparable to the dissected cadavers in a one-to-one manner.

To our knowledge this is the first study comparing several imaging techniques of routine clinical use, i.e. ultrasound, radiography, computed tomography and magnetic resonance imaging, in embalmed cadavers. CT and MRI have been identified as techniques resulting in the best image quality, although logistics and price of imaging exceeds ultrasound or plain X-ray.

### Ultrasound

Ultrasound, a routinely used bedside technique in clinical medicine, is not suitable to scan intraabdominal and retroperitoneal organs in embalmed cadavers due to severe artifacts. These do not even allow the standard measurements of organ size. The commonly used embalming fluid, containing small amounts of formalin, not only causes a significant swelling of soft tissues [4,8], but also produces artifacts, that severely impair image quality [9]. The major problem seems to be the production of gas, which has been described by Saranteas et al. [16] and Tsui et al. [17]. On the other hand, de Maeseneer et al. [18] showed that ultrasound quality of muscles of the hand in embalmed body donors was still better than ultrasound images of fresh cadavers due to a homogenising effect of the embalming fluid. Benkhadra et al. [14] showed similar results for imaging muscles, nerves and organs of the cervical region and ultrasound images of nerves and vessels of the lower extremity in embalmed cadavers even present images similar in quality to those taken from the living [19]. Further studies should investigate if those images offer useful information for teaching in the dissection course. Summing up, despite the theoretical advantage of this technique the rather poor imaging quality of intraabdominal and retroperitoneal organs disable the use of ultrasound in embalmed bodies. Based on the results of our study so far, ultrasound is not reasonable to be introduced to the dissection course as desired. Nevertheless, it remains the imaging tool of choice for 'living anatomy’ courses on life subjects.

### Radiography

Plain x-ray techniques are preferred for a quick overview of skeletal structures as well as fluid accumulation in the lungs and distribution of gas content in the gut. In embalmed bodies this technique has a very limited use, even though mobile x-ray machines would allow imaging of human body donors right in the anatomical institute. In addition to severe gas artifacts, x-ray imaging was limited by the sagging of abdominal organs towards the spinal column due to the position of the body donors during fixation and storage. This prohibited the penetration of radiation through the whole body. Our results are in contrast to the report of De Crop et al. [13], who found thorax images of embalmed cadavers to be equivalent to those of patients. This might be due to the difference in embalming solution and/or procedure. Nevertheless, radiography can be useful for upper and lower limb and joint imaging of embalmed human bodies. This needs to be further investigated.

### Computed tomography

The quality of computed tomography (CT) images was slightly impaired by gas artifacts as previously described [9,15]. Due to perimortal changes and fixation artifacts, the lungs and other parenchymal organs were viewable on CT scans, but the precise detection of organ details was difficult. In the present study skeletal structures could clearly be detected, enabling CT imaging to be a suitable method to teach students and to identify pathological changes of the skeletal system.

### MRI

The homogeneous appearance of embalmed tissues seems to be the disadvantage for magnetic resonance imaging (MRI). The fixation affects the water mobility and signal intensities of tissues, thus making the discrimination of tissues difficult [8,19,20]. Nevertheless, the authors declare, that for teaching purposes the image quality is still good enough because the topographical anatomy is still clearly observable [4,8]. In this study, the images of shoulder and knee joints were of very good quality.

## Conclusions

We conclude that CT and MRI are suitable methods for imaging embalmed human bodies. In our view, the high logistic effort for those two techniques is justified given the superior image quality in comparison to ultrasound and plain x-ray. Moreover, while ultrasound requires a skilled examiner and a constant visual feedback guiding the position of the ultrasound probe, topography of CT scans and MRIs are easier to grasp. Moreover, both CT and MRI even allow the three-dimensional reconstruction of complex anatomical structures or pathological processes. Despite the involved cost and effort we are convinced that the preparation of structures that underwent imaging just prior to anatomical dissection causes a great gain in knowledge and represents a valuable addition to early medical education.

Future studies need to clarify if the logistic and human investment into new teaching tools is well perceived and evaluated.

### Limitations

This study only included a small number of human cadavers. For further studies we suggest the investigation of a larger sample of body donors, especially to identify possible gender differences. In this study we focused on the comparison of the feasibility of imaging techniques in embalmed cadavers for teaching purposes. The results reflect an only small part of possible scientific questions. For future studies a wider variety of aims and objectives, e.g. the comparison of sensitivity and specificity of the different imaging techniques for certain measurements, should be investigated to maximise the information of collected data. Furthermore, to accurately compare the image quality across all modalities, all four imaging techniques should be conducted with all body donors. We also recommend the comparison of different embalming techniques to see if they influence the image quality of the four modalities in different ways.

## Competing interests

The authors declare that they have no competing interests.

## Authors’ contributions

RS participated in the design of the study, coordinated the study procedure, participated in the interpretation of data, and drafted the manuscript. DS carried out the x-ray, CT, and MRI imaging, participated in the interpretation of data, and revised the manuscript critically. AR carried out the ultrasound imaging, participated in the interpretation of data, and revised the manuscript critically. JK participated in the design of the study, participated in the interpretation of data, and drafted the manuscript. MH participated in the design of the study, carried out the ultrasound imaging, participated in the interpretation of data, and revised the manuscript critically. HK participated in the design of the study, coordinated the study procedure, participated in the interpretation of data, and revised the manuscript critically. All authors read and approved the final manuscript.

## Authors’ information

RS, M.D., is a resident physician at the Department of Anatomy and Cell Biology, Faculty of Medicine, Martin-Luther-University Halle-Wittenberg, Halle (Saale), Germany. She teaches gross anatomy, neuroanatomy, embryology, and histology to first and second year medical students.

DS, M.D., is a senior physician at the Department of Radiology, Faculty of Medicine, Martin-Luther-University Halle-Wittenberg, Halle (Saale), Germany. He teaches radiology to third year medical students. Furthermore he is the head of the Dorothea Erxleben SkillsLab of the Faculty of Medicine, Martin-Luther-University Halle-Wittenberg, Halle (Saale), Germany.

AR, M.D., is a senior physician at the Department of Internal Medicine, Division of Nephrology and Hypertension, Medical School Hannover, Germany. He teaches internal medicine, physical examination and nephrology at different time points during the model medical curriculum HannibaL (Hanoverian integrated, job-oriented and adaptive curriculum) at Hannover Medical School, as well as ultrasound techniques to residents and fellows.

JK, M.D., is an associate professor at the Department of Internal Medicine, Division of Nephrology and Hypertension, Medical School Hannover, Germany. He teaches internal medicine, physical examination and nephrology at different time points during the model medical curriculum HannibaL (Hanoverian integrated, job-oriented and adaptive curriculum) at Hannover Medical School.

MH, M.D., is a senior physician at the Department of Internal Medicine, Division of Nephrology and Hypertension, Medical School Hannover, Germany. He teaches internal medicine, physical examination and nephrology at different time points during the model medical curriculum HannibaL (Hanoverian integrated, job-oriented and adaptive curriculum) at Hannover Medical School, as well as ultrasound techniques to residents and fellows.

HK, M.D., is a professor at the Department of Anatomy and Cell Biology, Faculty of Medicine, Martin-Luther-University Halle-Wittenberg, Halle (Saale), Germany. She teaches gross anatomy, embryology, and histology to first and second year medical students.

## Pre-publication history

The pre-publication history for this paper can be accessed here:

http://www.biomedcentral.com/1472-6920/13/143/prepub

## Supplementary Material

Additional file 1 **Intravascular gas artifacts in the arcuate arteries and in the surrounding tissue of the kidney severely limit the use of ultrasound in embalmed human cadavers.** Movement of air bubbles can be seen in several small renal blood vessels, which did not dissolve by tissue compression with the ultrasound transducer. Click here for file
